# Non-invasive ^19^F MR spectroscopy of 5-fluorocytosine to 5-fluorouracil conversion by recombinant *Salmonella* in tumours

**DOI:** 10.1038/sj.bjc.6601345

**Published:** 2003-10-28

**Authors:** T Dresselaers, J Theys, S Nuyts, B Wouters, E de Bruijn, J Anné, P Lambin, P Van Hecke, W Landuyt

**Affiliations:** 1 Biomedical NMR Unit, KU Leuven, Herestraat 49, 3000 Leuven, Belgium; 2 MAASTRO Lab/GROW, University Maastricht, PO BOX 616, 6200 MD Maastricht, The Netherlands; 3 Experimental Radiobiology/LEO, KU Leuven, Gasthuisberg-CDG, Herestraat 49, 3000 Leuven, Belgium; 4 Laboratory Experimental Oncology, KU Leuven, Gasthuisberg-CDG, Herestraat 49, 3000 Leuven, Belgium; 5 Laboratory of Bacteriology, KU Leuven, Rega Institute, Minderbroederstraat 10, 3000 Leuven, Belgium

**Keywords:** ^19^F MRS, 5-fluorouracil, cytosine deaminase, xenograft human tumour, mouse, recombinant bacteria

## Abstract

The aim of this study was to evaluate the applicability of fluorine-19 magnetic resonance spectroscopy (^19^F MRS) for monitoring *in vivo* the conversion of 5-fluorocytosine (5-FC) to 5-fluorouracil (5-FU) after using an attenuated *Salmonella* Typhimurium strain recombinant to provide cytosine deaminase (TAPET-CD). The ^19^F MRS measurements were done on mice bearing the human colon tumour xenograft (HCT116). The intratumoural conversion is greater when TAPET-CD/5-FC is delivered intratumourally (*i.tu.*) than when TAPET-CD is delivered intravenously *(i.v.)* and 5-FC intraperitoneally *(i.p.)*. Repeat measurements of the same tumour also yielded important information on the tumour colonization by TAPET-CD through the correlated 5-FC to 5-FU conversion efficacy. The *in vivo* MRS spectra were confirmed by *in vitro* ^19^F MRS of perchloric acid extracts of the tumour tissue. No 5-FU metabolites were detectable *in vivo* in the tumours. However, the *in vitro* measurements revealed, besides 5-FC and 5-FU, the presence of small amounts of catabolites. Finally, spectra obtained *in vitro* from liver extracts of tumour-bearing mice treated *i.tu.* with TAPET-CD/5-FC showed no 5-FU and only little amounts of catabolites. Our data illustrate most importantly the potential of ^19^F MRS to monitor biologically-based treatments involving cytosine deaminase.

New developments for cancer treatment are allowing individualized and biologically-based therapies. As part of these developments one needs to accurately define both physical and biological characteristics of tumours, and to monitor the efficacy of treatment on an individual basis. Techniques such as computed tomography (CT) or magnetic resonance imaging (MRI) have been shown extremely useful in defining the physical limits of the tumour. Progress has also been made with regards to the evaluation of biological parameters of the tumour microenvironment (eg, hypoxia) and dynamic monitoring of therapy-induced changes (eg, 5-fluorouracil (5-FU) chemotherapy). Yet, further progress is of crucial importance for the evaluation of new biologically-based therapies that may be more tumour specific than traditional therapies, and which necessitate a stringent individualized follow-up. Technologies that are being explored in this context are positron emission tomography (PET) and magnetic resonance spectroscopy (MRS). The application of biological imaging methodologies is essentially non-invasive, and thus biopsies before and during the treatment do not need to be taken, leaving the tumour microenvironment undisturbed. Furthermore, these methods allow potential screening of tumours at many sites of the body and enable the direct comparison with normal tissues.

The use of MRS to non-invasively assess biochemical properties of tumours (eg, pH and lactate), and to evaluate concentrations and pharmacology of fluorinated chemotherapeutics has been reported. Compounds such as 5-FU lend themselves to this methodology because fluorine-19 (^19^F) has high sensitivity (83% relative to ^1^H), and because only negligible amounts of ^19^F are normally present in the body. 5-FU is a well-known chemotherapeutic agent, and has been evaluated along with the various metabolites. Although 5-FU measurements are often done in plasma samples, studies indicate that plasma levels do not reflect the intratumoural activity of 5-FU (Finan *et al*, 1987; Hull *et al*, 1988; Findlay *et al*, 1996). Using ^19^F MRS, 5-FU and metabolites could be detected directly in the tumour and in the liver, in both preclinical animal investigations as well as in patient studies of systemically delivered 5-FU (Stevens *et al*, 1984; Wolf *et al*, 1990; reviewed in Martino *et al*, 2000). Studies in animals and patients suggest that the trapping of 5-FU and the amount of anabolites in the tumour correlate with response and may yield prognostic information on treatment outcome (McSheehy *et al*, 1989, 1992; Wolf *et al*, 1990, 1998; Findlay *et al*, 1993; Presant *et al*, 1994; Schlemmer *et al*, 1999). Furthermore, in MRS the area of the spectral peak is proportional to the concentration of the corresponding metabolite (absolute concentrations require calibration). Although PET is more sensitive than MRS, the former technique lacks the capacity to distinguish *in vivo* between the prodrug, the drug and the different metabolites.

Others and we have been investigating a new biologically-based cancer therapy involving the cytosine deaminase (CDase) enzyme/prodrug system. The non-mammalian enzyme CDase converts the non-toxic 5-FC to the cytostatic drug (5-FU). Previous results showed that recombinant bacteria can be used as a delivery vector to transfer CDase selectively to the compromised intratumoural microenvironment. The tumour-selective applicability of this strategy has recently been demonstrated *in vitro* and *in vivo* with non-pathogenic clostridia (Minton *et al*, 1995; Fox *et al*, 1996; Theys *et al*, 2001) and attenuated *Salmonella* Typhimurium (*msbB*^−^, *purI*^−^ strain; Vion Pharmaceuticals Inc.) (Low *et al*, 1999; Lee *et al*, 2001; King *et al*, 2002; Mei *et al*, 2002). Since such bacteria-based vectors are tumour selective, the enzyme/prodrug system may provide maximal tumour dosing with prolonged drug presence and circumvent treatment-limiting side effects (reviewed by Aghi *et al*, 2000).

MRS has already been shown to be able to detect conversion of 5-FC to 5-FU in tumours in which cells have been engineered to express CDase or by using a monoclonal tumour targeting antibody-CDase conjugate (Aboagye *et al*, 1998; Stegman *et al*, 1999).

The objective of the present study is to demonstrate the applicability of ^19^F MRS to assess non-invasively biological therapies. Specifically, ^19^F MRS is applied in a situation in which the expression of the therapeutic gene, and thus the conversion efficacy, is expected to be dependent on the microenvironment and biological characteristics of the tumour. Attenuated *S.* Typhimurium engineered to express CDase (TAPET-CD) were investigated in a mouse-xenografted human tumour model.

Our results show that the selective intratumoural production of 5-FU by a biologically-based therapy such as the TAPET-CD/5-FC strategy, can be monitored using ^19^F MRS.

## MATERIALS AND METHODS

### *In vivo* tumour model

HCT116 human colon tumour was xenografted subcutaneous (*s.c.*) in nu/nu mice using 1 × 10^6^ cells per 100 *μ*l. The cells were inoculated into the lower flank. At the time of TAPET-CD injection and of ^19^F MRS, the tumour volumes were between 300 mm^3^ and 2000 mm^3^ (tumour volumes were measured with Vernier calipers in three orthogonal directions and calculated with the formula L × H × W × PI/6).

All animal experiments were approved by the Animal Ethics Committee of the University ‘KU Leuven’, and procedures were according to the guidelines defined by the UKCCCR (Workman *et al*, 1998).

### Bacterial strain and non-MRS evaluation

The bacteria used were attenuated *Salmonella* Typhimurium, recombinant for cytosine deaminase (coded VNP20047, provided for research by VION Pharm. Inc. New Haven CT 06511, and hereafter referred to as TAPET-CD). The bacteria were grown in liquid modified LB medium at 37°C to a given number that was determined by OD_600 nm_ measurements (see details in Mei *et al*, 2002).

Intratumoural (*i.tu.*) injections of 1 × 10^8^ to 3 × 10^8^ bacteria per 100 *μ*l were given in two directions which, considering the size of the tumour and the insertion depth of the needle, were mainly central. The TAPET-CD were injected at a very slow rate to avoid leakage of the suspension. The intravenous injections (tail vein) consisted of 5 × 10^5^ to 1 × 10^6^ bacteria. The time of bacterial injection was considered day 0.

To evaluate the intratumoural colonization of the TAPET-CD (*n*=7), 1 g samples were taken aseptically and randomly from tumours not used for *in vitro* MRS. The tissue samples were homogenized and suspended in 1 ml of saline. Bacterial colonization was subsequently quantified by serial dilution series in modified LB broth. All samples were kept at 37°C for 24 h, at which time the colony-forming units (cfu) per gram of tissue were determined.

The preparation of the bacterial lysates for *in vitro* analysis of CDase activity has been described previously (Theys *et al*, 2001). Briefly, aliquots of an exponentially growing TAPET-CD culture were taken at various optical densities (OD_600 nm_) and centrifuged. The pellets were washed, sonicated, and the lysates were incubated at 37°C for 24 h with a 10 mg/ml solution of 5-FC to which Tris HCl (1 M) was added.

### Preparation of tissue perchloric acid extracts (PCA extracts)

Animals were sacrificed by cervical dislocation and immediately afterwards the tumour and liver were removed and snap frozen in liquid nitrogen. The whole tumour or liver was homogenized at 0°C in 5 ml of cold HClO_4_ (1 M) per gram tissue. After homogenization, the sample was incubated on ice for 1 h followed by centrifugation for 15 min at 1000 ***g*** (0°C). The supernatant was decanted and neutralized with KOH (10 M) and KHCO_3_ (1 M). The resulting precipitate was discarded following a second centrifugation. The supernatant was subsequently freeze-dried and the lyophilizate was dissolved in 0.5 ml of a potassium phosphate buffer (pH 7.0; 1 M).

### *In vivo* and *in vitro* MRS measurements

*In vivo* ^19^F MRS experiments on mice bearing the HCT116 tumour were performed at 188 MHz in a Bruker Biospec (Karlsruhe, Germany), equipped with a horizontal 4.7 Tesla superconducting magnet with 30 cm bore, using a 10 mm transmit-receive surface coil. Prior to the measurement, the mice were anaesthetized with intraperitoneally (*i.p.*) injected sodium pentobarbital (1 *μ*l/g body weight Nembutal®, Sanofi, Belgium). After administration of the 5-FC (*i.tu.* 100 *μ*l of a 7.5 *μ*g/*μ*l solution in saline by one injection central to the tumour since multiple injections may lead to multiple peaks (Guerquin-Kern *et al*, 2000), or *i.p.* 300 mg/kg (ie, on average 9 mg per mouse); SIGMA, Belgium), the mice were placed on a Perspex plate such that the tumour was positioned directly above the circular surface coil. Following shimming on the water proton signal, serial ^19^F NMR spectra were acquired every 13 min during 1 to 2.5 h (90 degree pulse of 11 *μ*s, total repetition time (TR)=0.75 s, number of averages (NA)=1024, spectral width=27 kHz; acquisition size=2048 points; no proton decoupling). The signals were processed by zerofilling to 4096 points and exponential multiplication of 6 Hz.

Most ^19^F MRS measurements were performed at day 0 and day 1. Some animals were also evaluated 1 or 2 weeks after the injection of TAPET-CD. Before a new injection of 5-FC was given, a spectrum was first recorded to determine whether there were still signals present from the previous injection (24 h earlier).

The chemical shift of the 5-FU resonance was set to 0 p.p.m. The 5-FC signal was observed around 1.2 p.p.m. The signal positions were verified with the *i.tu.* injection of 5-FC (as before) and 5-FU (40 *μ*l of 50 mg/ml Fluroblastine®, Pharmacia, Belgium) in tumour-bearing mice treated with TAPET-CD and were also correlated to the ^1^ H chemical shift of the tumour H_2_O resonance. A spherical external reference sample (diameter=7.5 mm, volume ∼100 *μ*l) containing 4-fluorobenzoic acid (FBA; ACROS, Belgium) in deuterated water saturated with chromium acetylacetonate (Cr(acac)_3_, ACROS, Belgium) was placed ∼9 mm under the coil to monitor stability of the MRS technique and coil load changes for the different mice/tumours under evaluation. Absolute concentrations were determined in a separate experiment using a cylindrical water phantom sample (diameter=25 mm, height=22 mm) containing 1.25 mM 5-FC, 2.5 mM 5-FU and 10 mM NaF.

During the *in vivo* ^19^F MRS measurements, the body temperature of the mice was kept at 36°C with the use of warm air ventilation in the magnet bore.

*In vitro* analysis of tissue extracts of TAPET-CD/5-FC-treated animals and *in vitro* analysis of lysates of TAPET-CD cultures were performed in a high resolution AMX 360 (8.4 Tesla) spectrometer (Bruker, Karlsruhe, Germany). Specific settings for the tissue extracts (278 K) were, TR=2.5 s, NA=3072 or 12288, ^1^H WALTZ-16-decoupling during the 1 s acquisition; and for the lysates (295 K), TR=2.5 s, NA=256, no proton decoupling. The tissue extracts were measured at 278 K to exploit the substantially shorter relaxation time at low temperature (typically a factor three) (Kamm *et al*, 1994). Absolute concentrations were determined using a phantom sample of 5-FU in water (2.8 mM, 500 *μ*l) with a coaxial FBA reference sample insert.

### Statistics

All results are expressed as averages ± SEM (with indication of the number of experiments). Linear regression analysis was performed where appropriate.

## RESULTS

### ^19^F MRS to evaluate *in vitro* the conversion capacity of the TAPET-CD

*In vitro* ^19^F MRS measurements of the conversion of 5-FC to 5-FU were initially performed in lysates from a series of TAPET-CD cultures to be used in the experiments. The conversion capacity increased as a function of TAPET-CD growth status (OD_600_) and was optimal when lysates were evaluated at the maximum of the growth phase (Table 1

**Table 1:** Conversion capacity of TAPET-CD as determined by *in vitro*^19^F MRS on lysates

**OD_600_**	**5-FC/total F (%)**	**5-FU/ total F (%)**
0.136	86	14
0.548	67	33
1.056	52	48
1.355	13	87
1.880	0	100

At several time-points in the TAPET-CD growth curve, lysates of the TAPET-CD culture were prepared and incubated with 5-FC for 24 h at 37°C. The optical density at 600 nm (OD_600_) is correlated (*R*^2^=0.945) with the conversion of 5-FC to 5-FU (relative to the total fluor content=5-FC+5-FU) as determined with *in vitro* ^19^F MRS (TR=2.5 s; NA=256; no ^1^H decoupling; *T*=295 K).

). This clearly demonstrated the relationship between the number of viable TAPET-CD and the 5-FU production (*R*^2^=0.945) and confirmed the quality of the TAPET-CD stock used for the *in vivo* experiments.

### *In vivo*^19^F MRS of *i.tu.* injected TAPET-CD and 5-FC

**Figure 1:**
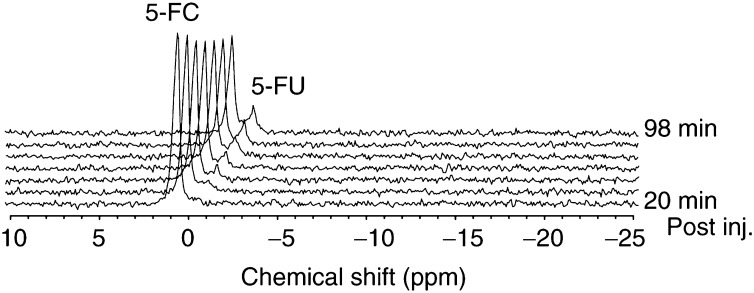
Serial ^19^F MRS spectra (13 min/spectrum; TR=0.75 s; NA=1024; LB=6 Hz; zerofilling to 4096 points) of a tumour in a mouse treated with the TAPET-CD system at day 0 and *i.tu.* injected with 5-FC (100 *μ*l of a 7.5 *μ*g/*μ*l solution in saline) at day 1.

We subsequently assessed the applicability of ^19^F MRS to evaluate *in vivo* non-invasively the conversion capacity of the TAPET-CD in the tumour microenvironment. The proof-of-principle was first demonstrated using HCT116 tumour-bearing mice and local *i.tu.* injection of both TAPET-CD and 5-FC. Figure 1

displays an example of these *in vivo* ^19^F MRS measurements performed 1 day after the TAPET-CD injection, starting immediately after the 5-FC injection. The 5-FC signal was maximal in the first acquisition that started within 10 min after the 5-FC administration (time needed for positioning of the animal, for shimming and for acquisition of a water signal used for calibration of the chemical shift), and declined slowly during the experimental time of up to 2 h. The 5-FU resonance peak became visible during the first 30 min of the measurement, increasing continuously thereafter as shown with the serial spectra. Both 5-FC and 5-FU resonances were measured with adequate spectral resolution. No clear catabolites or anabolites of 5-FU could be observed during the total experimental time.

Spectra recorded 24 h after the initial 5-FC to 5-FU conversion measurement, showed complete absence of fluorine signals. Conversion of 5-FC to 5-FU was however again detectable in these tumours following a repeat 5-FC *i.tu.* injection.

**Figure 2:**
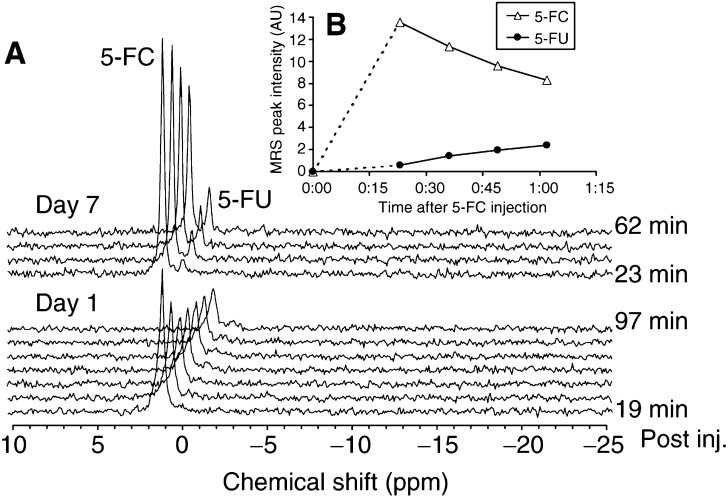
(**A**) ^19^F MRS study involving a repeat evaluation of a mouse treated with an *i.tu*. injection of the TAPET-CD system at day 0 followed with, in both cases, an *i.tu*. inoculation of 5-FC (100 *μ*l of 7.5 *μ*g/*μ*l) 6 to 10 min before start of the MRS acquisition (13 min/spec): lower group of spectra obtained at day 1, upper group at day 7. (**B**) Evolution of the MRS signal area of 5-FC and 5-FU in the HCT116 tumour at day 7 for the mouse described in A.

Intratumoural conversion of 5-FC to 5-FU was however not always present at day 1 following the *i.tu*. TAPET-CD injection. We hypothesized that this was the result of insufficient bacterial colonization in these tumours. We therefore repeated the ^19^F MRS analysis at a later time point without any additional administration of TAPET-CD. A representative example of such a measurement is illustrated in Figure 2A

. While very little 5-FC to 5-FU conversion was detectable at day 1 after the TAPET-CD was locally inoculated, a substantial conversion was measured during the measurement on day 7. For both time-points (day 1 and day 7), the ^19^F MRS analysis was started within 10 min after the *i.tu.* 5-FC injection. Figure 2B shows the time course for 5-FC and 5-FU derived from the top set of spectra in Figure 2A. The time course was similar for all experiments (slopes were −6.1±0.7 A.U./h for 5-FC and 2.0±0.4 A.U./h for 5-FU, based on linear regression; *n*=6). These data highlight the importance of repeat measurements for detecting the presence of TAPET-CD conversion efficacy during the tumour colonization period.

### *In vivo*^19^F MRS of *i.v.* injected TAPET-CD and *i.p.* 5-FC

**Figure 3:**
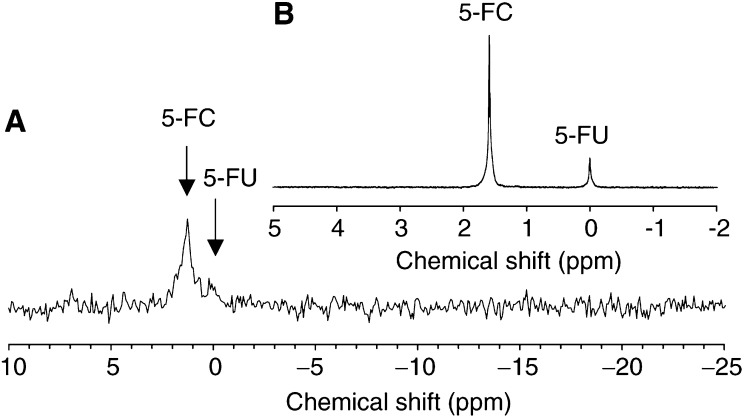
(**A**) *In vivo* ^19^F MRS spectrum obtained after 2 weeks (20 min/spectrum; TR=0.75 s; NS=1536) of a mouse treated with an *i.v.* injection of the TAPET-CD system (day 0) followed with an *i.p.* 5-FC injection (300 mg/kg). Spectrum obtained 2.5 h after 5-FC inoculation. (**B**) *In vitro* ^19^F MRS spectra of the perchloric acid extract of the whole tumour from the mouse in Figure 3A, snap-frozen immediately after the *in vivo* evaluation (TR=2.5 s; NA=12228; ^1^H decoupling).

The potential clinical applicability of TAPET-CD/5-FC involves not only *i.tu.* application but also the systemic delivery of the enzyme/prodrug system. Intratumoural production of 5-FU following *i.v.* injection of TAPET-CD and *i.p.* injection of 5-FC (300 mg/kg) was measured *in vivo* in the HCT116 colon tumour (Figure 3A

). The 5-FC concentration in the tumours following *i.p.* injection was comparable to the *i.tu*. administration (3-4 mM). A clear conversion of 5-FC to 5-FU was seen with this systemic application of TAPET-CD/5-FC, although the 5-FU signals were less than with the *i.tu.* delivery route (compare Figure 1 and Figure 3A).

Because of the reduced 5-FU signal, the conversion quality was further analysed and confirmed with *in vitro* high resolution ^19^F MRS (8.4 Tesla) of extract preparations from these tumours. Intratumoural concentrations of 5-FU were about 0.5±0.2 mM (*n*=6) for the *i.tu.* and 0.15±0.07 mM (*n*=7) for the systemically TAPET-CD *plus* 5-FC treated tumours respectively. A representative example of this analysis (Figure 3B) illustrates the efficacy of the TAPET-CD/5-FC conversion to 5-FU in this *i.v.*/*i.p.* administration modality.

**Figure 4:**
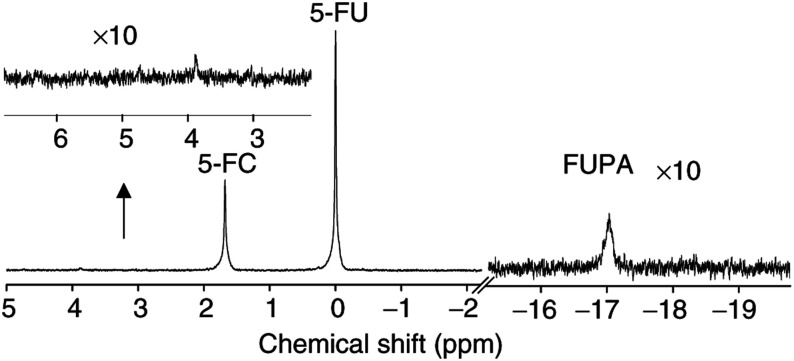
*In vitro* ^19^F MRS spectrum (TR=2.5 s; NA=12228; ^1^H WALTZ-16 decoupling) of the perchloric acid extract of the whole tumour shown in Figure 2, snap-frozen 2 h 47 min after the *in vivo* evaluation. A large conversion of 5-FC to 5-FU is visible in agreement with the large delay between *in vivo* ^19^F MRS and span freezing. Only small anabolite (∼4 p.p.m.) and catabolite signals (∼−17 p.p.m.) are observed.

Within the total *in vivo* experimental time, no metabolic activity was observed in the tumours, ie, the concentrations of the catabolites and anabolites of 5-FU were below the detection threshold of the *in vivo* ^19^F MRS. Using the much more sensitive *in vitro* MRS on tumour extracts, we observed a small catabolite signal around −17 p.p.m. (*i*.*tu.* 0.1±0.1 mM (*n*=4), *i.p.* 0.025±0.025 mM (*n*=5)) and in some cases also a very small anabolite signal between 3.5 and 5.5 p.p.m. (∼0.015 mM) (an example is shown in Figure 4

).

Those tumours, which were not further investigated with *in vitro* MRS, were analysed for bacterial colonization. Levels of 5 × 10^8^±2 × 10^8^ cfu per gram tissue were found.

### *In vitro*^19^F MRS of liver PCA extracts of mice treated with TAPET-CD/5-FC

**Figure 5:**
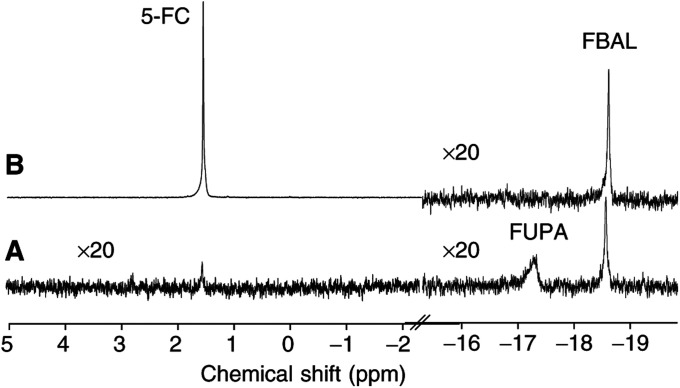
*In vitro* ^19^F MRS spectra (TR=2.5 s; NA=12228; ^1^H WALTZ-16 decoupling) of the perchloric acid extract of the whole liver: (**A**) The liver of a mouse (Figure 2) treated *i.tu.* with TAPET-CD on day 0 and injected *i.tu.* with 5-FC on day 1 and 7, snap-frozen 2 h 50 min after the *in vivo* evaluation (ie, about 4 h after the 5-FC administration). (**B**) The liver of a mouse treated *i.v.* with TAPET-CD (day 0) and *i.p.* with 5-FC (day 14), snap-frozen immediately after the *in vivo* evaluation (ie, about 2 h after the 5-FC administration).

It is obviously important to evaluate normal tissue in parallel with tumour tissue, as both ultimately determine the therapeutic window of treatment. Besides the perchloric acid extracts of tumours, also extracts of snap-frozen livers were thus analysed with *in vitro* ^19^F MRS. In all cases where conversion to 5-FU was detected in the tumour (both after 5-FC *i.p.* or *i.tu.*), small catabolite signals of *α*-fluoroureido-*β*-propionic acid (FUPA) at −17.3 p.p.m. and *α*-fluoro-*β*-alanine (FBAL) at −18.6 p.p.m. were observed in the liver extracts (Figure 5

). Although the conversion to 5-FU that was observed in the tumour after the *i.tu*. TAPET-CD/5-FC application was stronger than after the systemic route, the catabolite levels in the liver were similar for the *i.tu.* method (0.05±0.04 mM (*n*=3)) and the systemic administration (0.09±0.02 mM (*n*=6)).

## DISCUSSION

In the present ^19^F MRS study we demonstrate the non-invasive detection of the dynamic production of 5-FU from 5-FC in the human HCT116 colon tumour xenograft following the use of cytosine deaminase- (CDase-) recombinant *S.* Typhimurium (TAPET-CD). The results show intratumoural conversion of 5-FC to 5-FU within 30 min which proceeded throughout the MRS measurement time extending up to 1.5–2 h after the 5-FC injection. This suggests that continued intratumoural production of 5-FU is greater than the diffusion of 5-FU from the tumour into the systemic circulation of the animal. The high resolution *in vitro* ^19^F MRS analysis of the snap-frozen whole tumours confirmed the *in vivo* results. At the end of the MRS measurement, the *i.tu.* 5-FU signal corresponded to about 0.5±0.2 mM.

With investigations involving the injection of *i.v*. TAPET-CD and *i.p*. 5-FC, we measured an intratumoural 5-FU concentration of about 0.15±0.07 mM two hours post 5-FC injection. Although this is more than three times less than what is found after an *i.tu.* injection (0.5±0.2 mM), this still represents about 20 *μ*g/ml 5-FU (based on 300 mg/kg 5-FC given *i.p.* in the present experiments). Lambin and colleagues discussed the potential of the bacteria-based CDase/5-FC strategy to improve the therapeutic outcome from the combination with radiotherapy (Lambin *et al*, 2000). Based on published data they estimated that an intratumoural production of 0.6–0.9 *μ*g/ml of 5-FU would lead to a radiosensitization factor of 1.1–1.2 when combined with standard 2 Gy radiotherapy schedule. Therefore the 5-FU levels obtained with the present bacterial CDase/5-FC system should lead to a highly significant radiosensitization.

The *in vivo* ^19^F MRS indicated the lack of 5-FU metabolites in tumours. Yet, the *in vitro* measurements of PCA extracts of the same tumours enabled the detection of small amounts of catabolites and sometimes anabolites. The formation of fluoronucleosides and fluoronucleotides from 5-FU is known to be a slow process and may also depend on the type of tumour under investigation. The concentration of the free anabolites could on the other hand remain below the sensitivity of the ^19^F MRS, although cytostatic activity is present, a possibility discussed by McSheehy and Griffiths (1989).

We investigated *in vitro* the extracts of several snap-frozen livers from TAPET-CD/5-FC treated tumour-bearing mice that presented with a strong intratumoural 5-FU signal. This ^19^F MRS analysis revealed the absence of 5-FU and only small amounts of catabolites in the liver, at least up to 2 h after the *in vivo* measurements. Our results agree with the observations made by King *et al*, (2002) using high-performance liquid chromatography. The difference between the small catabolite levels in our study and the large levels usually found with conventional 5-FU chemotherapy, is obvious for two reasons: (1) it is expected that the ‘leakage’ of 5-FU from the tumour microenvironment into the systemic circulation is poor (eg, indirectly shown by Wallace *et al*, 1994), and (2) this type of conversion occurs mainly in the liver and little FBAL enters the systemic circulation (discussed by eg, Prior *et al*, 1990).

The difference in spectral quality for mice treated with *i.p. versus i.tu.* 5-FC injection is, besides the conversion quality, also partially related to the broader linewidths that are observed after an *i.p.* injection. This increases the overlap of the 5-FC peak with the 5-FU peak and decreases the signal-to-noise ratio. For this reason the 5-FU signal was most clearly observed when the 5-FC signal had significantly decreased (about 2.5 h after injection).

The ^19^F MRS measurements allowed us to gain a better understanding of some of the characteristics of the TAPET-CD/5-FC treatment strategy. First, a period ranging from 1 to 7 days seems necessary for the bacteria to establish and proliferate selectively in the tumours and express sufficient levels of CDase. This agrees with our previous work using the TAPET-CD/5-FC, in which the rat rhabdomyosarcoma tumour model was used (Mei *et al*, 2002). Using *in vitro* analysis of bacterial lysates, we also clearly demonstrated the positive correlation between the number of TAPET-CD and the amount of 5-FC that is converted to 5-FU for a given incubation period. Second, the TAPET-CD persist in the tumours without the need for a repeat injection, similar to the findings of King *et al*, (2002) and Mei *et al*, (2002). Indeed, in the present study, the production of 5-FU after the *i.p.* 5-FC administration was measurable even at 14 days after the injection of the bacteria. Third, intertumoural variation in the production of 5-FU *in vivo* was present. This can likely not be explained on the basis of differences in TAPET-CD colonization of the tumours. Indeed, the analysis for intratumoural presence revealed colonization levels of 5 × 10^8^±2 × 10^8^ cfu per gram of tumour tissue. However, tumours are inherently morphological heterogeneous, a fact which may partly explain differences of drug levels (as discussed by Kamm *et al*, 1996).

## CONCLUSION

The present results are to our knowledge the first demonstration of the applicability of ^19^F MRS to monitor *in vivo* the intratumoural activity of CDase-recombinant bacteria. This suggests that MRS may be useful for monitoring therapy with TAPET-CD/5-FC since it allows repetitive examinations without the need to interfere with the microenvironment. The use of non-pathogenic strains of bacteria to transfer effector genes and therapeutic proteins selectively to the tumour microenvironment is being intensively investigated (Minton *et al*, 1995; Zheng *et al*, 2000; Theys *et al*, 2001; King *et al*, 2002; Liu *et al*, 2002). Based on the promising preclinical *in vitro* and *in vivo* data, including the positive safety profile, the application of the attenuated *Salmonella* strain (both *i.tu.* and *i.v.* administration) is being tested in clinical phase I studies (Cunningham and Nemunaitis, 2001; Toso *et al*, 2002). From the perspective of the broader application of the novel anti-cancer treatment (eg, optimisation of protocol, combining with radiotherapy or chemotherapy), the non-invasive MR methodology thus offers a significant advance for individual guidance and longitudinal monitoring of treatment.
